# Exposure to Intrapartum Epidural Analgesia and Risk of Autism Spectrum Disorder in Offspring

**DOI:** 10.1001/jamanetworkopen.2022.14273

**Published:** 2022-05-26

**Authors:** Malia S. Q. Murphy, Robin Ducharme, Steven Hawken, Daniel J. Corsi, William Petrcich, Darine El-Chaâr, Lise Bisnaire, Daniel I. McIsaac, Deshayne B. Fell, Shi Wu Wen, Mark C. Walker

**Affiliations:** 1 Clinical Epidemiology Program, Ottawa Hospital Research Institute, Ottawa, Ontario, Canada; 2 School of Epidemiology and Public Health, University of Ottawa, Ottawa, Ontario, Canada; 3 ICES, Ottawa, Ontario, Canada; 4 Children’s Hospital of Eastern Ontario Research Institute, Ottawa, Ontario, Canada; 5 Better Outcomes Registry & Network (BORN) Ontario, Children’s Hospital of Eastern Ontario, Ottawa, Ontario, Canada; 6 Department of Obstetrics and Gynecology, University of Ottawa, Ottawa, Ontario, Canada; 7 Department of Obstetrics, Gynecology and Newborn Care, The Ottawa Hospital, Ottawa, Ontario, Canada; 8 Department of Anesthesiology and Pain Medicine, University of Ottawa, Ottawa, Ontario, Canada; 9 International and Global Health Office, University of Ottawa, Ottawa, Ontario, Canada

## Abstract

**Importance:**

There is conflicting evidence on the association between intrapartum epidural analgesia and risk of autism spectrum disorder (ASD) in offspring.

**Objective:**

To evaluate the association between intrapartum epidural analgesia and the risk of ASD in offspring.

**Design, Setting, and Participants:**

This population-based cohort study was conducted in Ontario, Canada, using the health and administrative records of singleton live births by vaginal delivery between April 1, 2006, and March 31, 2014. Neonates with less than 24 weeks' gestation or weighing less than 500 g were excluded. Offspring were followed up from 18 months of age until ASD diagnosis, loss to follow-up, or the end of the study (December 31, 2020), whichever occurred first. Exposure, covariate, and outcome data were obtained using provincial health administrative databases.

**Exposures:**

Any intrapartum exposure to epidural or combined spinal-epidural analgesia.

**Main Outcomes and Measures:**

The primary outcome was ASD diagnosis after 18 months of age. Inverse probability of treatment weighting (IPTW) of Cox proportional hazards regression models was used to estimate the hazard ratio (HR) of intrapartum epidural analgesia and ASD in offspring. Offspring head injury was used as a control outcome. Models were adjusted for maternal sociodemographic factors, health behaviors, and medical and obstetrical history as well as labor, delivery, and offspring characteristics. Post hoc analyses included restriction to term neonates, a conditional within-mother analysis, exclusion of records with concomitant intrapartum pain management exposures, a complete case analysis, use of an alternative ASD definition, and estimation of the average treatment effect in the treated group.

**Results:**

Among the 650 373 mother-offspring pairs included in the study, 418 761 (64.4%) were exposed to intrapartum epidural analgesia. The mean (SD) maternal age at delivery was 29.7 (5.5) years; the offspring had a mean (SD) gestational age at delivery of 39.1 (1.6) weeks and included 329 808 male newborns (50.7%). The exposed and unexposed groups were similar in all maternal and newborn characteristics after IPTW (standardized difference <0.10). Autism spectrum disorder was diagnosed in 7546 offspring (1.8%) of mothers who received intrapartum epidural analgesia (incidence rate, 18.8 [95% CI, 18.4-19.3] per 10 000 person-years) compared with 3234 offspring (1.4%) who were unexposed (incidence rate, 14.4 [95% CI, 13.9-14.9] per 10 000 person-years). The crude HR for ASD associated with intrapartum epidural analgesia was 1.30 (95% CI, 1.25-1.36), and the IPTW-adjusted HR was 1.14 (95% CI, 1.08-1.21). Results did not qualitatively differ in post hoc analyses.

**Conclusions and Relevance:**

Results of this study showed that intrapartum epidural analgesia was associated with a small increase in risk for ASD in offspring. The biological plausibility of this association, however, remains unclear, and the finding must be interpreted with caution.

## Key Points

**Question:**

Is there an association between administration of intrapartum epidural analgesia and risk of autism spectrum disorder (ASD) in offspring?

**Findings:**

In this cohort study of 650 373 mother-offspring pairs in Ontario, Canada, intrapartum epidural analgesia was statistically associated with a small increase in risk of ASD in offspring. The association remained after a series of post hoc analyses.

**Meaning:**

Findings from this study suggest an association between intrapartum epidural analgesia and ASD in offspring, but the biological plausibility of this association remains unclear, and these findings must be interpreted with caution.

## Teaser

This cohort study examines the incidence of autism spectrum disorder at age 18 months among the offspring of mothers who received epidural or combined spinal-epidural analgesia during labor.

## Introduction

Epidural analgesia is a commonly used pharmacological intervention for labor analgesia. In Canada, more than 60% of the 275 000 women who delivered vaginally from 2018 to 2019 received an epidural.^1^ In the US, more than 80% of women in labor receive epidural analgesia, spinal analgesia, or combined spinal-epidural analgesia for a vaginal birth.^2^ Patients who receive epidural and spinal-epidural analgesia during labor report higher satisfaction levels and lower pain scores compared with those who receive other forms of intrapartum pain management, such as nitrous oxide or opioids.^3^ Factors associated with the choice to deliver with epidural analgesia include higher educational level, higher household income, and nulliparity.^4^ However, rates of epidural use are greatly affected by health care accessibility and are higher in urban areas, in larger obstetrical centers, and among women receiving care from an obstetrician.^5^ Although complications can occur, epidural and spinal-epidural analgesia are widely considered safe for both mother and offspring.^3,6,7^

Although the effectiveness of epidural analgesia for mitigating pain during labor and delivery has been well documented, the long-term outcomes associated with its use remain uncertain. In general, there are limited and conflicting data on the magnitude and direction of association between intrapartum pain management and the risk of neurodevelopmental outcomes in offspring who were exposed to epidural analgesia.^8,9,10,11,12,13,14,15,16,17^ Recent data from some population-based cohorts have suggested an association between intrapartum epidural analgesia and offspring autism spectrum disorder (ASD), with adjusted hazard ratios (aHRs) ranging from 1.08 (95% CI, 1.02-1.15) to 1.37 (95% CI, 1.23-1.53).^16,17,18^ In contrast, other studies have found no statistical association, with aHRs of 1.05 (95% CI, 0.98-1.11) and 1.08 (95% CI, 0.97-1.20).^11,15^ All of these findings must be interpreted cautiously. Methodological challenges inherent in this research area include residual confounding from unmeasured sociodemographic and clinical parameters, exposure and outcome misclassification, and the inability to account for parental genetics and other risk factors for neurodevelopmental sequelae.^19^ Statements issued from professional societies representing the obstetric, pediatric, and anesthesiology communities maintain that there is insufficient evidence to alter the current anesthetic care practices offered to obstetric patients.^20,21,22^

In this study, we sought to evaluate the association of intrapartum epidural analgesia with the risk of ASD in offspring. We performed robust adjustments for confounding factors and used data from Canada’s largest provincial birth registry.

## Methods

This retrospective, population-based cohort study was reviewed and approved by the Ottawa Health Science Network Research Ethics Board and the ICES Privacy Office. We obtained data from ICES, an independent nonprofit research institute whose legal status under Ontario’s health information privacy law allows it to collect and analyze health care and demographic data without informed consent for the purpose of health system evaluation and improvement.^23^ We followed the Strengthening the Reporting of Observational Studies in Epidemiology (STROBE) reporting guideline.

### Study Population and Data Sources

We identified all in-hospital singleton live births by vaginal delivery in Ontario, Canada, between April 1, 2006, and March 31, 2014. Neonates who were born at less than 24 weeks’ gestation or weighed less than 500 g were excluded. To facilitate follow-up, we excluded neonates whose mothers were not continuously eligible for coverage under the publicly funded Ontario Health Insurance Plan (OHIP) within the 2 years leading up to and including the offspring’s birth date; also excluded were newborns who were not eligible for OHIP coverage within 7 days of birth. Records with missing or invalid values for date of birth, offspring sex, gestational age at delivery, mode of delivery, and birth weight were excluded. Offspring who died or were lost to follow-up before 18 months of age were also excluded.

The cohort was derived from the Better Outcomes Registry & Network (BORN) Ontario data set, which includes maternal demographic and pregnancy characteristics, health behaviors, labor and delivery details, such as use of pain management, and pregnancy and newborn outcome information for nearly all births (>99%) in Ontario.^24^ Records were linked with the MOMBABY data set at ICES along with other health administrative data sets (eTable 1 in the Supplement) to verify and/or ascertain additional exposure, covariate, and outcome data. Data sets were linked using unique encoded identifiers and were analyzed at ICES.^23^

### Exposures

The primary exposure was any intrapartum exposure to epidural or combined spinal-epidural analgesia (exposed group). Epidural exposure was first identified from the BORN Ontario data set, which routinely captures information on pain management during the hospital admission for delivery, including epidural, spinal and pudendal blocks, local and general anesthesia, nitrous oxide, narcotics, and nonpharmacologic methods. We also searched the Canadian Institute for Health Information Discharge Abstract Database at ICES for the intervention anesthetic technique code 3 or C (epidural or combined spinal and epidural anesthetic) and the Canadian Classification of Health Interventions code 5.LD.20.HA-P1 (intrapartum pharmacotherapy during active labor using anesthetic agents with a percutaneous approach).^25^ After supplementing with the Discharge Abstract Database records, we assumed that the remaining records for which intrapartum epidural administration was not noted were unexposed (unexposed group).

### Outcomes

The primary outcome was ASD diagnosis after 18 months of age.^26,27^ Follow-up began at age 18 months and continued until ASD was documented, loss to follow-up (because of death or migration), or the end of the study period (December 31, 2020), whichever occurred first. Offspring were classified as having ASD if their health records included the *International Statistical Classification of Diseases and Related Health Problems, Tenth Revision (ICD-10)* diagnosis code F84.x for any single hospital discharge, emergency department visit, or outpatient surgery or the OHIP diagnostic code 299.x for ASD 3 times in 3 years.^28^ This algorithm has been validated using health administrative data in Ontario and was chosen from a range of other algorithms to maximize both the positive predictive value (PPV) and sensitivity: sensitivity of 50.0% (95% CI, 40.7%-88.7%), specificity of 99.6% (95% CI, 99.4%-99.7%), PPV of 56.6% (95% CI, 46.8%-66.3%), and a negative predictive value of 99.4% (95% CI, 99.3%-99.6%). Accordingly, the positive likelihood ratio was 125, and the negative likelihood ratio was 0.50.

Offspring head injury, a commonly used control outcome in vaccine research, was used to assess the validity of the findings. This outcome was broadly defined using *ICD-10* codes S00 to S09 to capture a wide range of superficial or minor wounds and major traumas.

### Covariates

Covariates of interest were prespecified according to clinical and epidemiological knowledge. Covariate data included maternal sociodemographic information at delivery (age, neighborhood income quintile, and urban or rural place of residence), the number of maternal all-cause health care encounters in the 5 years before delivery, obstetrical characteristics (parity, previous cesarean delivery, conception type, and antenatal health care practitioner type), health conditions in pregnancy (preexisting or gestational diabetes and preexisting hypertension or hypertensive disorders arising during pregnancy), and self-reported smoking and/or drug use in pregnancy. Covariates also included offspring and delivery characteristics, such as birth year, birth season, induction or augmentation of labor, oxytocin exposure, spontaneous or assisted vaginal delivery, gestational age, birth weight, sex, Apgar score at 5 minutes, small for gestational age (<3rd or <10th percentile for birth weight^29^), large for gestational age (>90th percentile for birth weight^29^), neonatal intensive care unit admission for more than 24 hours, maternal intention to breastfeed, and hospital characteristics (number of obstetrical beds and level of maternity care^30^). Although data on race and ethnicity are available from BORN Ontario, maternal race and ethnicity were not included as covariates in this study because of the large proportion of missing data.

Multiple imputation was used to impute missing values for covariates. A total of 208 840 records had at least 1 missing covariate and required imputation. The proportion of records that required imputation of at least 1 covariate was 33.2% (76 857 of 231 612) among the unexposed group and 31.5% (131 983 of 418 761) among the exposed group. Ten imputed data sets were created using a fully conditional specification approach. The extent of missing covariate values for exposed and unexposed groups before multiple imputation is provided in eTable 2 in the Supplement.

### Statistical Analysis

All analyses were conducted using SAS Enterprise Guide, version 7.15 (SAS Institute). Descriptive analyses were conducted to compare mother-offspring pairs who received intrapartum epidural analgesia with pairs who did not receive epidural analgesia. Continuous variables were described using means (SDs) or medians (IQRs). Categorical variables were described using frequencies and percentages. A standardized difference of 0.10 or greater was considered to be indicative of covariate imbalance between the exposed and unexposed groups.

Time-to-event analyses were conducted using Cox proportional hazards regression models, with offspring age in days as the underlying time scale, to generate HRs with 95% CIs for exposed vs unexposed mother-offspring pairs. We used robust sandwich estimators to account for multiple deliveries to 1 mother. Follow-up for time-to-event outcomes began on the day the offspring turned 18 months of age for the primary outcome of ASD diagnosis. Those who did not experience the events of interest were censored at death, loss to follow-up, or last date of available data (March 31, 2020), whichever occurred first. Proportional hazards assumptions in models were assessed using log-log survival curves and residual plots.

Inverse probability of treatment weighting (IPTW) was used in Cox proportional hazards regression models to adjust for potential confounding. We developed a multivariable logistic regression model to calculate a propensity score for each mother-offspring pair, representing the estimated probability of exposure to intrapartum epidural analgesia. Covariates and covariate categories that were included in the propensity score model were established a priori according to clinical knowledge and a literature review for factors associated with intrapartum analgesia use and/or risk of ASD (eTable 3 in the Supplement).^31^ Adjusted HRs were estimated from weighted Cox proportional hazards regression models using IPTW in each of the 10 data sets created from multiple imputation. The resulting parameters and SEs were pooled across the 10 imputed data sets to construct a single adjusted estimate and 95% CI. The eMethods in the Supplement provide the SAS codes used for multiple imputation, IPTW modeling, and Cox proportional hazards regression models.

We performed a series of post hoc analyses to assess the implications of the study design for the observed outcomes. First, we restricted the cohort to term neonates (≥37 weeks’ gestation). Second, to address the possible genetic role in ASD risk, we conducted a within-mother analysis by fitting a conditional model to account for mothers with multiple offspring in the cohort. We also fit within-mother conditional models in subsets of the cohort: (1) restricted to offspring with 1 or more siblings and (2) restricted to siblings with discordant exposure statuses (1 unexposed and 1 exposed). Third, to explore the direct role of intrapartum epidural analgesia in ASD risk, the exposed group was restricted to those with only intrapartum epidural analgesia exposure, and those with evidence of exposure to other pain management options (including spinal-epidural combinations) were removed from the analysis.

Fourth, we restricted the cohort to include only those with complete data before multiple imputation (ie, complete case analysis). Fifth, we applied an alternative ASD outcome definition that maximized the specificity or PPV at the cost of some sensitivity, and we mirrored the approach used by previous investigators.^11,15^ Offspring aged 18 months or older with at least 1 relevant *ICD-10* code (F84.x) for any hospital discharge, emergency department visit, or outpatient surgery was considered to have ASD. This algorithm has been validated in Ontario health administrative data and was found to have the following performance measures: sensitivity of 20.5%, specificity greater than 99.9%, PPV of 85.2%, and negative predictive value of 99.1%.^28^ Sixth, we modified the weights used in the Cox proportional hazards regression models to estimate the average treatment effect in the treated group.^32^

## Results

A total of 728 224 live singleton births by vaginal delivery occurred in Ontario hospitals during the study period. After exclusions, the cohort included 650 373 mother-offspring pairs, with 418 761 (64.4%) exposed to intrapartum epidural analgesia and 231 612 (35.6%) unexposed (Figure). The mean (SD) maternal age at delivery was 29.7 (5.5) years, and there were 320 565 female (49.3%) and 329 808 male (50.7%) newborns with a mean (SD) gestational age at delivery of 39.1 (1.6) weeks. The median (IQR) follow-up time for the primary outcome was 9.7 (7.7-11.8) years. A total of 501 offspring (0.1%) died, and 23 467 (3.6%) were lost to follow-up for various reasons, including migration.

**Figure.**
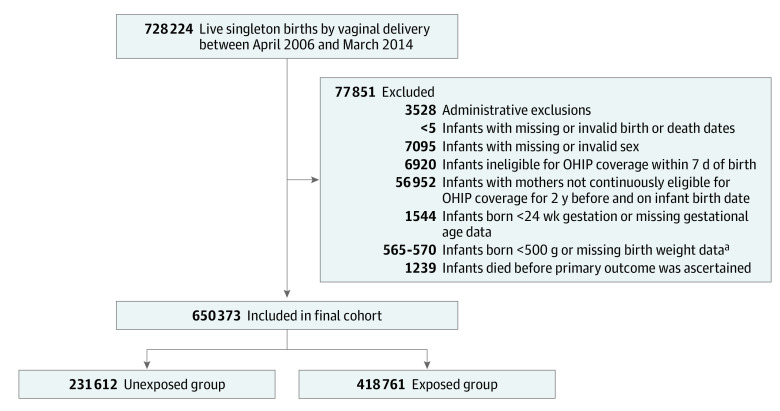
Patient Flowchart OHIP indicates Ontario Health Insurance Plan. ^a^A range is presented in keeping with ICES policies to prevent back calculation of suppressed numbers. In this case, a range is presented to prevent back calculation of the <5 infants with missing or invalid birth or death dates.

We observed an imbalance between the 2 groups in several baseline covariates before propensity score weighting (Table 1). Compared with mothers in the unexposed group, mothers who received intrapartum epidural analgesia were less likely to live in neighborhoods with a lower median income, were more likely to live in an urban setting, and were more likely to have a higher number of health care encounters in the 5 years before giving birth. Mothers in the exposed group were also more likely to be nulliparous, receive antenatal care from an obstetrician, and deliver in a hospital with a higher number of obstetrical beds and higher level of maternity care. In addition, mothers who received intrapartum epidural analgesia were more likely to have undergone induction or augmentation of labor than those who did not receive epidural analgesia and, accordingly, were more likely to receive oxytocin. After multiple imputation and weighting, we found that covariate distribution was balanced between the 2 exposure groups, with standardized differences of less than 0.10 for all covariates (Table 1).

**Table 1.** Maternal, Offspring, and Hospital Characteristics at Delivery

Characteristic	Intrapartum epidural exposure					
	Unweighted, No. (%)			IPTW, %		
	Unexposed group (n = 231 612)	Exposed group (n = 418 761)	Standardized difference	Unexposed group (n = 231 612)	Exposed group (n = 418 761)	Standarized difference
**Maternal characteristics**						
Age at delivery, mean (SD), y	29.7 (5.5)	29.7 (5.5)	0.002	29.7 (9.4)	29.7 (6.8)	0.004
Age at delivery, y						
<20	8906 (3.8)	17 111 (4.1)	0.01	4.3	3.9	0.02
20-24	32 746 (14.1)	56 809 (13.6)	0.02	14.2	13.7	0.02
25-29	67 026 (28.9)	120 475 (28.8)	0.004	28.6	28.9	0.006
30-34	77 420 (33.4)	144 437 (34.5)	0.02	32.6	34.6	0.04
≥35	45 514 (19.7)	79 929 (19.1)	0.01	20.2	19.0	0.03
Neighborhood income quintile						
1 (lowest)	56 033 (24.2)	82 962 (19.8)	0.11	21.7	21.6	0.002
2	47 291 (20.4)	81 927 (19.6)	0.02	20.2	20.1	0.003
3	46 391 (20.0)	86 600 (20.7)	0.02	20.4	20.6	0.003
4	45 604 (19.7)	93 300 (22.3)	0.06	21.1	21.3	0.006
5 (highest)	34 532 (14.9)	72 397 (17.3)	0.06	16.5	16.4	0.004
Missing	1761 (0.8)	1575 (0.4)	0.05	NA	NA	NA
Urban residence						
Yes	93 734 (83.6)	388 236 (92.7)	0.28	89.4	89.7	0.01
No	37 753 (16.3)	30 358 (7.2)	0.28	10.6	10.3	0.01
Missing	125 (0.1)	167 (0)	0.007	NA	NA	NA
No. of health care encounters in the 5 y before delivery, mean (SD)	50.6 (29.1)	55.1 (30.5)	0.15	53.8 (50.5)	53.7 (37.4)	0.002
Nulliparity	66 143 (28.6)	207 518 (49.6)	0.44	41.3	42.4	0.02
Previous cesarean delivery	7642 (3.3)	13 881 (3.3)	0.001	96.4	96.5	0.006
Spontaneous conception	191 258 (82.6)	343 164 (81.9)	0.02	98.1	98.1	0.003
Diabetes in pregnancy^a^	14 497 (6.3)	29 905 (7.1)	0.04	7.5	7.4	0.001
Hypertension in pregnancy^b^	18 388 (7.9)	42 045 (10.0)	0.07	10.3	10.0	0.007
Smoking in pregnancy^c^	29 968 (12.9)	46 633 (11.1)	0.06	12.6	12.6	0.001
Drug use in pregnancy^d^	3505 (1.5)	4948 (1.2)	0.03	1.4	1.4	0.002
Antenatal care practitioner^e^						
Obstetrician (all or partial care)	138 755 (59.9)	323 937 (77.4)	0.38	75.4	75.8	0.007
Family physician, nurse, or nurse practitioner only	40 342 (17.4)	52 450 (12.5)	0.14	15.0	14.9	0.003
Midwife	39 357 (17.0)	20 379 (4.9)	0.4	9.5	9.4	0.007
Missing	13 158 (5.7)	21 995 (5.3)	0.02	NA	NA	NA
**Delivery characteristics **						
No. of obstetrical beds at hospital in birth fiscal year, mean (SD)	22.2 (14.6)	29.1 (16.9)	0.44	26.5 (29.5)	26.6 (20.2)	0.009
Level of maternity care						
1	45 303 (19.6)	37 859 (9.0)	0.3	13.9	13.7	0.005
2	148 398 (64.1)	287 577 (68.7)	0.1	70.5	70.8	0.007
3	21 521 (9.3)	74 445 (17.8)	0.25	15.6	15.5	0.004
Not otherwise specified	≤5 (0)	≤5 (0)	0.01	0	0	0.004
Missing	16 386 (7.1)	18 880 (4.5)	0.11	NA	NA	NA
Birth year						
2006	27 410 (11.8)	46 225 (11.0)	0.03	11.4	11.4	0.001
2007	30 351 (13.1)	50 896 (12.2)	0.03	12.7	12.6	0.004
2008	30 603 (13.2)	52 542 (12.5)	0.02	12.9	12.8	0.002
2009	28 046 (12.1)	50 247 (12.0)	0.003	12.2	12.1	0.006
2010	29 706 (12.8)	53 183 (12.7)	0.004	12.9	12.8	0.003
2011	29 069 (12.6)	54 763 (13.1)	0.02	13.0	12.9	0.002
2012	28 359 (12.2)	55 449 (13.2)	0.03	12.5	12.8	0.009
2013	28 068 (12.1)	55 456 (13.2)	0.03	12.4	12.7	0.009
Birth season						
Fall (September 22-December 20)	55 116 (23.8)	99 782 (23.8)	0.001	23.8	23.8	<0.001
Spring (March 21-June 20)	59 029 (25.5)	106 326 (25.4)	0.002	25.3	25.4	0.002
Summer (June 21-September 21)	62 567 (27.0)	113 004 (27.0)	0.001	27.0	27.0	0.001
Winter (December 21-March 20)	54 900 (23.7)	99 649 (23.8)	0.002	23.8	23.8	0.001
Induction or augmentation of labor	110 745 (47.8)	324 687 (77.5)	0.65	66.5	67.2	0.02
Oxytocin for labor management	50 726 (21.9)	251 872 (60.1)	0.84	44.9	46.6	0.03
**Offspring characteristics**						
Gestational age at delivery, mean (SD) wk	39.0 (1.7)	39.1 (1.6)	0.08	39.04 (2.8)	39.1 (2.0)	0.01
Birth weight, mean (SD), g	3386.5 (519.4)	3404.3 (496.9)	0.03	3392.8 (862.8)	3394.9 (625.7)	0.00
Female sex	114 818 (49.6)	205 747 (49.1)	0.009	49.0	49.3	0.005
Male sex	116 794 (50.4)	213 014 (50.9)	0.01	51.0	50.7	0.01
Apgar score <4 at 5 min	270 (0.1)	529 (0.1)	0.003	0.2	0.1	0.03
SGA (3rd percentile)^f^	1844 (0.8)	3050 (0.7)	0.01	0.8	0.8	<0.001
SGA (10th percentile)^f^	8109 (3.5)	14 173 (3.4)	0.01	3.5	3.4	0.004
LGA (90th percentile)^f^	49 710 (21.5)	87 964 (21.0)	0.01	21.1	21.1	0.001
NICU admission >24 h	17 607 (7.6)	38 522 (9.2)	0.06	8.8	8.7	0.004
Maternal intention to breastfeed	216 372 (93.4)	391 347 (93.5)	0.001	97.2	97.2	<0.001

Abbreviations: IPTW, inverse probability of treatment weighting; LGA, large for gestational age; NA, not applicable; NICU, neonatal intensive care unit; SGA, small for gestational age.

^a^ Diabetes in pregnancy included diabetes diagnosis before pregnancy or during the index pregnancy (gestational diabetes).

^b^ Hypertension in pregnancy included hypertension before pregnancy (chronic hypertension) or hypertensive disorders in the index pregnancy, including gestational hypertension, preeclampsia, eclampsia, and HELLP (hemolysis, elevated liver enzymes, and low platelets) syndrome.

^c^ Smoking in pregnancy included smoking during the index pregnancy at any prenatal or delivery visit.

^d^ Drug use in pregnancy included use of any drugs before the index pregnancy, including cocaine, gas or glue, hallucinogens, cannabis, methadone, narcotics, opioids, or other drugs.

^e^ If a woman had a midwife in addition to other health care practitioners, she was assigned to the midwife group. The assumption was that most of the antenatal care would have been provided by the midwife.

^f^ Small for gestational age and large for gestational age were assigned using INTERGROWTH-21 Project categories.^29^

### Association Between Intrapartum Epidural Analgesia and ASD

A total of 10 780 offspring (1.7%) had a diagnosis of ASD after 18 months of age by the end of the follow-up period. The mean (SD) age at diagnosis of ASD was 4.9 (2.4) years in the exposed group and 4.6 (2.4) years in the unexposed group. Autism spectrum disorder was identified in 7546 offspring (1.8%) of mothers who received intrapartum epidural analgesia (incidence rate, 18.8 [95% CI, 18.4-19.3] per 10 000 person-years) and among 3234 offspring (1.4%) of mothers who did not receive intrapartum epidural analgesia (incidence rate, 14.4 [95% CI, 13.9-14.9] per 10 000 person-years). The crude HR for ASD associated with intrapartum epidural analgesia was 1.30 (95% CI, 1.25-1.36), which was attenuated but remained statistically significant after IPTW adjustment (aHR, 1.14; 95% CI, 1.08-1.21) (Table 2).

**Table 2.** Association Between Exposure to Intrapartum Epidural Analgesia and ASD in Offspring

Outcome	Unexposed group		Exposed group		Crude HR (95% CI)	IPTW-adjusted HR (95% CI)
	No. with outcome/total No. (%)	Incidence rate per 10 000 person-years (95% CI)	No. with outcome/total No. (%)	Incidence rate per 10 000 person-years (95% CI)		
Primary outcome						
ASD^a^	3234/231 612 (1.4)	14.4 (13.9-14.9)	7546/418 761 (1.8)	18.8 (18.4-19.3)	1.30 (1.25-1.36)	1.14 (1.08-1.21)
Negative control outcome						
Head injuries^b^	72 791/231 612 (31.4)	403 (400.1-405.9)	132 245/418 761 (31.6)	409.8 (407.6-412.0)	1.01 (1.00-1.02)	1.01 (1.00-1.02)
Post hoc analyses						
Restricted to term offspring	2937/218 525 (1.3)	13.8 (13.3-14.3)	6973/ 397 935 (1.8)	18.3 (17.8-18.7)	1.31 (1.26-1.37)	1.14 (1.08-1.21)
Conditional within-mother analysis						
Offspring with or without siblings	3234/231 612 (1.4)	14.4 (13.9-14.9)	7546/418 761 (1.8)	18.8 (18.4-19.2)	1.20 (1.06-1.36)	1.15 (1.05-1.26)
Restricted to siblings	1508/120 610 (1.3)	13.1 (12.4-13.7)	3156/ 194 068 (1.6)	16.6 (16.0-17.2)	1.20 (1.06-1.36)	1.14 (1.04-1.26)
Restricted to siblings with different exposure statuses	532/41 117 (1.3)	14.2 (13.0-15.4)	682/41 493 (1.6)	16.0 (14.8-17.3)	1.20 (1.06-1.36)	1.17 (1.06-1.28)
Restricted to offspring with epidural exposure only	3234/231 612 (1.4)	14.4 (13.9-14.9)	4902/271 283 (1.8)	18.9 (18.4-19.4)	1.30 (1.25-1.36)	1.14 (1.08-1.21)
Complete case analysis	2213/154 755 (1.4)	15.3 (14.6-15.9)	5162/286 778 (1.8)	19.4 (18.9-19.9)	1.26 (1.2-1.3)	1.12 (1.05-1.20).
Revised definition of ASD^c^	666/231 612 (0.3)	2.9 (2.7-3.2)	1643/418 761 (0.4)	4.1 (3.9-4.3)	1.38 (1.26-1.51)	1.19 (1.06-1.34)
ASD, using modified weights to estimate ATT	3234/231 612 (1.4)	14.4 (13.9-14.9)	7546/418 761 (1.8)	18.8 (18.4-19.2)	1.30 (1.25-1.36)	1.14 (1.07-1.22)

Abbreviations: ASD, autism spectrum disorder; ATT, average treatment effect on treated; HR, hazard ratio; IPTW, inverse probability of treatment weighting.

^a^ ASD diagnosis after 18 months of age. ASD was defined either as at least 1 *International Statistical Classification of Diseases and Related Health Problems, Tenth Revision (ICD-10)* diagnosis code for any single hospital discharge, emergency department visit, or outpatient surgery or as 3 Ontario Health Insurance Plan diagnostic codes for ASD in 3 years, unless otherwise specified.

^b^ Defined by *ICD-10* diagnosis codes S00 to S09 inclusive. These head injuries include superficial head injury (S00), open head wound (S01), skull and facial bone fracture (S02), dislocation and sprain of joints and ligaments of the head (S03), cranial nerve injury (S04), eye and orbit injury (S05), intracranial injury (S06), crushing head injury (S07), avulsion and traumatic amputation of part of the head (S08), and other and unspecified head injuries (S09).

^c^ ASD diagnosis after 18 months of age. Autism spectrum disorder was defined as at least 1 *ICD-10* diagnosis code for any single hospital discharge, emergency department visit, or outpatient surgery.

### Findings From Post Hoc and Control Outcome Analyses

In post hoc analyses, restricting the cohort to term offspring, conducting a within-mother analysis, restricting the cohort to mother-offspring pairs with exposure to epidurals only, conducting a complete case analysis, using an alternative case-finding definition for ASD, and performing an average treatment effect on treated analysis did not substantively change the findings (Table 2). For example, use of the alternative case-finding algorithm was associated with increased effect estimate (aHR, 1.19; 95% CI, 1.06-1.34).

A total of 205 036 offspring (31.5%) had a documented head injury during the follow-up period. The IPTW-adjusted HR for head injuries associated with exposure to intrapartum epidural analgesia was 1.01 (95% CI, 1.00-1.02) (Table 2).

## Discussion

In this population-based cohort study, we found that offspring who were exposed to intrapartum epidural analgesia had a 14% relative increase in risk of ASD diagnosis after 18 months of age (aHR, 1.14; 95% CI, 1.08-1.21), compared with those without epidural exposure after adjustment for maternal (demographic, socioeconomic, and obstetrical) and neonatal confounders. Although these findings suggest an association between epidural use and ASD, the results must be interpreted with caution, and the methodological approaches used across studies must be carefully considered. The biological plausibility of this finding remains unclear, and residual confounding may account for the observations in this study.

The results were consistent with 2 recent population-based retrospective cohort studies that found an association between intrapartum epidural analgesia and offspring risk of ASD, with a study from Southern California reporting an aHR of 1.37 (95% CI, 1.23-1.53)^16^ and the other study from British Columbia, Canada, reporting an aHR of 1.09 (95% CI, 1.00-1.15).^17^ In contrast, 2 large analyses found no association between use of labor epidural and ASD risk in offspring after accounting for a wide range of sociodemographic, preexisting maternal factors as well as pregnancy-related and perinatal factors: 1 study from Manitoba, Canada, reported an aHR of 1.08 (95% CI, 0.97-1.20),^11^ and 1 national analysis from Denmark reported an aHR of 1.05 (95% CI, 0.98-1.11).^15^ In addition, a nationwide cohort study of public and private US insurance records reported a small risk increase (aHR, 1.08; 95% CI, 1.02-1.15).^18^ Its authors also reported the pooled effect estimate from a meta-analysis they conducted of published cohort studies (aHR, 1.10; 95% CI, 1.06-1.13).^18^

Despite the use of similar case-finding algorithms, covariates, and post hoc analyses, the differences in findings across the Canadian and Danish studies highlight the challenges of comparing the results from disparate administrative data sources. Regional variations in how ASD diagnoses are captured and the quality and quantity of exposure and covariate data are all likely to change the magnitude and direction of reported associations. In the current study, labor epidural analgesia was not associated with all-cause head injuries in offspring. This finding may provide some reassurance about the validity of the approach used, but it does not confirm a causal association between intrapartum epidural exposure and ASD.

### Strengths and Limitations

This study has some strengths. The study had a large population size, which was derived from BORN Ontario, the largest birth registry in Canada. Although the study period and duration were similar to those of recently published population-based studies (all lasting 7-14 years from 2000-2016) on this topic,^11,15,16,17^ the sample size available for the present analysis was substantially larger and the rate of epidural use in this cohort was consistent with estimates across Canada (approximately 60%).^33^ Furthermore, BORN Ontario includes extensive information on preexisting maternal conditions as well as obstetrical, delivery, and newborn outcomes that enabled us to account for a wide range of potential confounders, including pregnancy-related complications and other pharmacological interventions for labor augmentation. Use of propensity score methods created covariate balance between the 2 exposure groups, removing the impact of these measured covariates in this analysis. The proportion of missing data was low for most variables, and the use of multiple imputation, a widely accepted method for addressing missing data, reduced the potential for bias associated with missing information for confounding variables.

This study also has several limitations. First, although the cohort had complete exposure ascertainment, the variables used to ascertain labor pain management have not been previously validated. The BORN Ontario registry has a rigorous data quality framework to validate data at the time of entry, for completion and accuracy. Internal and external audits of other BORN Ontario variables have demonstrated higher than 90% agreement with clinical records.^34,35,36^ In this study, 90% of offspring in the exposed group had mothers with intrapartum epidural administration that was registered in both BORN Ontario and health administrative data at ICES. Thus, although exposure misclassification is a concern, we mitigated this issue by verifying the exposure status using multiple population-level data sets. Second, case-finding algorithms to identify individuals with ASD in health administrative data are subject to misclassification bias, and the findings must be interpreted cautiously.^28,37^ The algorithm we used in the main analysis was derived from Ontario health administrative data and was selected to maximize both PPV and sensitivity.^28^ Although misclassification is likely to be nondifferential and to bias the results toward the null, a PPV of 56.6% suggests that as many as 44% of ASD cases in this cohort may be false positives. We attempted to address misclassification in a post hoc analysis by using an alternative case-finding algorithm that maximized PPV (85.2%)^28^; subsequently, the magnitude of the effect estimate increased from 1.14 (95% CI, 1.08-1.21) to 1.19 (95% CI, 1.06-1.34).

Third, the findings may not be generalizable to other settings because the environmental factors that affect neurodevelopment, diagnoses, and physician billing practices are likely to vary by jurisdiction. Even within Ontario, the algorithm we used favors identification of older offspring, those seen within specialized clinical settings, and those with more complex presentation.^28^ Fourth, the propensity score methods established balance in measured covariates between exposure groups, but we cannot guarantee balance in unmeasured covariates or covariates that were not included in the propensity score model. There are likely other mediators and confounders that we were unable to account for with the available data. Notable factors that were not examined in this study included length of labor as well as duration and dose of exposure to epidural analgesia. Although we conducted a within-mother analysis to address heritable factors, we were unable to directly account for paternal factors. There are hundreds of gene variants that have been found to be associated with ASD, and it is unlikely that all inherited factors associated with predisposition to ASD were accounted for.^38^ In addition, we did not account for prenatal exposure to environmental toxins, which have recently been linked to adverse neurodevelopmental outcomes, including ASD.^39^

## Conclusions

In this population-based cohort study of live births in Ontario, Canada, administration of labor epidural analgesia was associated with a small increase in ASD risk in offspring. The findings were insufficient to demonstrate causation, and given the methodological limitations inherent in observational studies, we must interpret these findings with caution. Although the epidemiological association between intrapartum epidurals and ASD in offspring has been examined widely, little attention has been paid to its biological plausibility. This topic warrants further investigation given that interest in this topic among researchers, health care practitioners, and patients is likely to persist.

## Notes

Supplement. **eTable 1.** ICES Datasets Used in This Analysis **eTable 2.** Distribution of Missing Covariate Values Between Exposed and Unexposed Offspring Before Multiple Imputation **eMethods.** SAS Code Used for Multiple Imputation, IPTW Modeling and Cox Regression **eTable 3.** Covariates and Covariate Categories Used in Multivariable Logistic Regression Models for Propensity Score Development
