# Supplementary material for: Exposure to Intrapartum Epidural Analgesia and Risk of Autism Spectrum Disorder in Offspring
Source: JAMA Netw Open. 2022 May 26;5(5):e2214273. doi: 10.1001/jamanetworkopen.2022.14273 (PMC9136620; doi:10.1001/jamanetworkopen.2022.14273)
Supplement: Supplement. — eTable 1. ICES Datasets Used in This Analysis eTable 2. Distribution of Missing Covariate Values Between Exposed and Unexposed Offspring Before Multiple Imputation eMethods. SAS Code Used for Multiple Imputation, IPTW Modeling and Cox Regression eTable 3. Covariates and Covariate Categories Used in Multivariable Logistic Regression Models for Propensity Score Development [file jamanetwopen-e2214273-s001.pdf]

Supplemental Online Content

Murphy MSQ, Ducharme R, Hawken S, et al. Exposure to intrapartum epidural analgesia and risk of autism spectrum disorder in offspring. *JAMA Netw Open.* 2022;5(5):e2214273. doi:10.1001/jamanetworkopen.2022.14273

eTable 1. ICES Datasets Used in This Analysis

eTable 2. Distribution of Missing Covariate Values Between Exposed and Unexposed Offspring Before Multiple Imputation

eMethods. SAS Code Used for Multiple Imputation, IPTW Modeling and Cox Regression

eTable 3. Covariates and Covariate Categories Used in Multivariable Logistic Regression Models for Propensity Score Development

This supplemental material has been provided by the authors to give readers additional information about their work.

© 2022 Murphy MSQ et al. *JAMA Network Open.*

eTable 1. ICES Datasets Used in This Analysis

Dataset	Description	Use
Better Outcomes Registry & Network (BORN) Ontario	Includes maternal demographic data, and information on maternal medical and obstetrical histories, pregnancy complications and delivery and newborn outcomes.	Information on exposure status, baseline characteristics and obstetrical/delivery and newborn covariate data.
MOMBABY	An ICES derived dataset that links inpatient admission records of delivering mothers and their newborns	Verification of eligible maternal and newborn records
Ontario Health Insurance Plan (OHIP)	Includes all physician billing claims for inpatient visits, ambulatory visits, consultations and procedures	Primary and secondary outcomes, mothers' contact with the health care system within the 5 years prior to delivery
Registered Persons Database (RPDB)	Includes information on all individuals registered under the Ontario publicly funded health insurance plan (OHIP)	Ascertainment of birth dates, sex, death dates, place of residence
Canadian Institute for Health Information's (CIHI) Discharge Abstract Database (DAD)	Includes administrative, clinical and demographic information on hospital discharges (including deaths and transfers)	Primary and secondary outcomes, supplemental data for exposure, mothers' contact with the health care system within the 5 years prior to delivery
CIHI's National Ambulatory Care Reporting System (NACRS)	Includes data on hospital- and community-based ambulatory care (including day surgery, outpatient, community based clinics and emergency departments)	Primary and secondary outcomes, mothers' contact with the health care system within the 5 years prior to delivery
CIHI's Same Day Surgery (SDS)	Includes data on individuals receiving same-day/out-patient surgeries	Primary and secondary outcomes, mothers' contact with the health care system within the 5 years prior to delivery
Emergency Claims Database (ERCLAIMS)	Includes data on OHIP billings by emergency physicians and thus comprises data on patient visits that occurred in the Emergency Department.	Primary and secondary outcomes, mothers' contact with the health care system within the 5 years prior to delivery
Ontario Census Area Profiles (CENSUS)	Includes individual and house-hold level socioeconomic data from the national short-form census questionnaire distributed to Ontario households every 10 years	Information on place of residence (urban vs rural) and area-level income and education quintiles based on maternal postal code at the time of delivery
Information about Ontario Health Care Institutions (INST)	Includes information about all Ontario health care institutions funded by the Ministry of Health and Long-Term Care (MOHLTC);	Information on delivery hospital's level of maternal and newborn care
Ontario Diabetes Dataset (ODD)	An ICES derived dataset that captures incident cases of diabetes	Information on maternal diabetes status prior to pregnancy
Ontario Hypertension Dataset (HYPER)	An ICES derived dataset that captures incident cases of hypertension	Information on maternal hypertension status prior to pregnancy

© 2022 Murphy MSQ et al. *JAMA Network Open.*

eTable 2. Distribution of Missing Covariate Values Between Exposed and Unexposed Offspring Before Multiple Imputation

	**Number and proportion of records with missing values, n(%)**	
	**Unexposed N=231,612**	**Exposed N=418,761**
Maternal age at delivery	0 (0)	0 (0)
Maternal neighbourhood income quintile	1,761 (0.8)	1,575 (0.4)
Maternal rural residence	125 (0.1)	167 (0.0)
Maternal health care encounters before delivery	0 (0)	0 (0)
Nulliparous	1,986 (0.9)	5,290 (1.3)
Previous cesarean	6,724 (2.9)	17,423 (4.2)
Spontaneous conception	37,998 (16.4)	67,458 (16.1)
Diabetes in pregnancy[a]	17,234 (7.4)	29,196 (7.0)
Hypertension in pregnancy[b]	15,411 (6.7)	24,901 (5.9)
Smoking in pregnancy[c]	15,426 (6.7)	28,913 (6.9)
Drug use in pregnancy[d]	17,201 (7.4)	28,571 (6.8)
Antenatal care practitioner type[e]	13,158 (5.7)	21,995 (5.3)
Number of obstetrical beds at delivery hospital in birth fiscal year	252 (0.1)	74 (0.0)
Maternity hospital level of caref	16,386 (7.1)	18,880 (4.5)
Birth year	0 (0)	0 (0)
Birth season	0 (0)	0 (0)
Induction or augmentation of labour	948 (0.4)	1,008 (0.2)
Oxytocin for labour management	0 (0)	0 (0)
Gestational age at delivery, weeks	0 (0)	0 (0)
Birthweight	0 (0)	0 (0)
Offpsring sex	0 (0)	0 (0)
Apgar score <4 at 5 min	1,698 (0.7)	2,428 (0.6)
Small for gestational age (3rd percentile)[f]	0 (0)	0 (0)
Small for gestational age (10th percentile)[f]	0 (0)	0 (0)
Large for gestational age (90th percentile)[f]	0 (0)	0 (0)
NICU admission >24 hr	0 (0)	0 (0)
Maternal intent to breastfeed	9,046 (3.9)	16,523 (3.9)

[a]Diabetes in pregnancy included maternal diabetes diagnosis prior to pregnancy or during the index pregnancy (gestational diabetes).

[b]Hypertension in pregnancy included maternal hypertension prior to pregnancy (chronic hypertension) or hypertensive disorders in the index pregnancy including gestational hypertension, pre-eclampsia, eclampsia, and HELLP.

[c]Smoking in pregnancy included maternal smoking in the index pregnancy at any prenatal or delivery visit.

[d]Drug use in pregnancy included any maternal use of drugs during the index pregnancy including cocaine, gas/glue, hallucinogens, cannabis, methadone, narcotics, opioids or other.

[e]If a woman had a midwife in addition to other health care practitioners, she was assigned to the midwife group. The assumption was that most of the antenatal care would have been provided by the midwife.

[f]Small for gestational age and large for gestational age were assigned based on INTERGROWTH-21 categories

© 2022 Murphy MSQ et al. *JAMA Network Open.*

eMethods. SAS Code Used for Multiple Imputation, IPTW Modeling and Cox Regression

```
*Key portions of SAS analysis programs - variable names have been
modified to better show what each is capturing;

*Imputation done using PROC MI;

proc mi data=epid_full nimpute=10 out=mi_epid_final seed = 382020536;
class
primary_outcome
exposed
baby_birth_year oxytocin
sex income_quintile rural reproductive_assistance antenatal_care
drug_use_in_pregnancy intention_to_breastfeed induced_augmented
hypertension_disorder_in_preg diabetes_in_pregnancy smoking_in_pregnancy
parity_category birth_season previous_caesarean nicu_admission sga3 sga10
lga90 hospital_level
;
var
primary_outcome
exposed
baby_birth_year oxytocin
sex income_quintile rural reproductive_assistance antenatal_care
drug_use_in_pregnancy intention_to_breastfeed induced_augmented
hypertension_disorder_in_preg diabetes_in_pregnancy
smoking_in_pregnancy parity_category birth_season previous_caesarean
nicu_admission sga3 sga10 lga90 hospital_level
age gest_age birth_weight apgar5 hospital_obstetrical_beds
natural_log_of_healthcare_use
;
fcs
logistic(reproductive_assistance antenatal_care hospital_level / likelihood
= augment link=glogit)
logistic(income_quintile rural drug_use_in_pregnancy
intention_to_breastfeed induced_augmented hypertension_disorder_in_preg
diabetes_in_pregnancy smoking_in_pregnancy parity_category
previous_caesarean / likelihood = augment link=logit)
regpmm (apgar5 hospital_obstetrical_beds);
run;
```

© 2022 Murphy MSQ et al. *JAMA Network Open.*

```
*Propensity score calculation and weighting;

*In the EFFECT statement below, the macro variable &pctls_ln_hcu.
contains a comma-delimited list of the following percentiles of the
values of the natural logarithm of the healthcare use variable (counting
health-care use prior to baseline):

  5th 27.5th 50th 72.5th and 95th;

*The BY variable _IMPUTATION_ labels the data as being part of imputed data
sets 1 to 10;

proc logistic data=mi_epid_final;
by _imputation_;
class
baby_birth_year oxytocin
sex income_quintile rural reproductive_assistance antenatal_care
drug_use_in_pregnancy intention_to_breastfeed induced_augmented
hypertension_disorder_in_preg diabetes_in_pregnancy
smoking_in_pregnancy parity_category birth_season previous_caesarean
nicu_admission sga3 sga10 lga90 hospital_level(ref='Level 1')
;
effect spl_hcu = spline(ln_hcu / naturalcubic basis=TPF(noint)
knotmethod=list(&pctls_ln_hcu.) );

model exposed(ref = '0') =
spl_hcu
baby_birth_year oxytocin
age gest_age birth_weight apgar5 hospital_obstetrical_beds
sex income_quintile rural reproductive_assistance antenatal_care
drug_use_in_pregnancy intention_to_breastfeed induced_augmented
hypertension_disorder_in_preg diabetes_in_pregnancy
smoking_in_pregnancy parity_category birth_season previous_caesarean
nicu_admission sga3 sga10 lga90 hospital_level;
output out = ps_scores_final pred = exp_propensity;
run;

*Calculation of weight values for IPTW;

data mi_working;
set  ps_scores_final;
if exposed eq 0 then iptw = 1/(1-exp_propensity);
else
if exposed eq 1 then iptw = 1/exp_propensity;
run;
```

© 2022 Murphy MSQ et al. *JAMA Network Open.*

```
*Primary outcome model;
*The BY variable _IMPUTATION_ labels the data as being part of imputed data
sets 1 to 10;
*The mother identifier is constant for each mother across their one or more
babies;
*fup_primary_outcome: time from index (birth) date to end of follow-up in
primary model;
*primary_outcome: captures whether event or censoring occurred;

proc phreg data=mi_working covs(aggregate);
by _imputation_;
class exposed(ref = first);
model fup_primary_outcome*primary_outcome(0) = exposed /
entrytime=start_of_outcome_detection_18mo;
weight iptw;
id mother_identifier;
hazardratio exposed/ diff=ref;
run;
```

© 2022 Murphy MSQ et al. *JAMA Network Open.*

eTable 3. Covariates and Covariate Categories Used in Multivariable Logistic Regression Models for Propensity Score Development

**Covariate**	**Covariate categories**
Maternal age at delivery, years	Modelled as a continuous variable
Maternal neighbourhood income quintile	Quintile 1 (lowest)<br>Quintile 2<br>Quintile 3<br>Quintile 4<br>Quintile 5 (highest)
Maternal rural place of residence	Yes<br>No
Number of maternal health care encounters in the 5 years before delivery	Modelled as a continuous variable using a 5-knot natural cubic spline
Parity	0 (nulliparous)<br>≥1
Previous cesarean	Yes<br>No
Conception type	Spontaneous<br>Intrauterine Insemination<br>Intrauterine Insemination/Assisted Reproductive Technologies<br>*In vitro* fertilization<br>*In vitro* fertilization – Intracytoplasmic sperm injection<br>*In vitro* fertilization and Intrauterine Insemination/Assisted Reproductive Technologies
Diabetes in pregnancy[a]	Yes<br>No
Hypertension in pregnancy[b]	Yes<br>No
Smoking in pregnancy[c]	Yes<br>No
Drug use in pregnancy[d]	Yes<br>No
Antenatal care practitioner type[e]	Obstetrician (all care or partial care)<br>NP, nurse, or family physician only<br>Midwife
Number of obstetrical beds at delivery hospital in birth fiscal year	Modelled as a continuous variable
Maternity hospital level of care	Level 1<br>Level 2

© 2022 Murphy MSQ et al. *JAMA Network Open.*

	Level 3<br>Not otherwise specified
Birth year	2006<br>2007<br>2008<br>2009<br>2010<br>2011<br>2012<br>2013
Birth season	Fall (22 Sept – 20 Dec)<br>Spring (21 Mar – 20 June)<br>Summer (21 June – 21 Sept)<br>Winter (21 Dec – 20 Mar)
Induction or augmentation of labour	Yes<br>No
Oxytocin for labour management	Yes<br>No
Gestational age at delivery, weeks	Modelled as a continuous variable
Birthweight, g	Modelled as a continuous variable
Offspring sex	Male<br>Female
Apgar score at 5 min	Modelled as continuous variable
Small for gestational age[f] (3rd percentile)	Yes<br>No
Small for gestational age[f] (10th percentile)	Yes<br>No
Large for gestational age[f] (90th percentile)	Yes<br>No
NICU admission >24 hr	Yes<br>No
Maternal intent to breastfeed	Yes<br>No

[a]Diabetes in pregnancy included maternal diabetes diagnosis prior to pregnancy or during the index pregnancy (gestational diabetes).

[b]Hypertension in pregnancy included maternal hypertension prior to pregnancy (chronic hypertension) or hypertensive disorders in the index pregnancy including gestational hypertension, pre-eclampsia, eclampsia, and HELLP.

[c]Smoking in pregnancy included maternal smoking in the index pregnancy at any prenatal or delivery visit.

[d]Drug use in pregnancy included any maternal use of drugs during the index pregnancy including cocaine, gas/glue, hallucinogens, cannabis, methadone, narcotics, opioids or other.

[e]If a woman had a midwife in addition to other health care practitioners, she was assigned to the midwife group. The assumption was that most of the antenatal care would have been provided by the midwife.

[f]Small for gestational age and large for gestational age were assigned based on INTERGROWTH-21 categories

© 2022 Murphy MSQ et al. *JAMA Network Open.*
